# Introducing 12 new dyes for use with oligonucleotide functionalised silver nanoparticles for DNA detection with SERS†

**DOI:** 10.1039/c8ra01998c

**Published:** 2018-05-15

**Authors:** L. Pala, S. Mabbott, K. Faulds, M. A. Bedics, M. R. Detty, D. Graham

**Affiliations:** Centre for Molecular Nanometrology, University of Strathclyde, Department of Pure and Applied Chemistry, Technology and Innovation Building 99 George Street Glasgow G1 1RD UK Duncan.Graham@strath.ac.uk; Department of Chemistry, University at Buffalo, The State University of New York New York 14260 USA

## Abstract

Oligonucleotide functionalised metallic nanoparticles (MNPs) have been shown to be an effective tool in the detection of disease-specific DNA and have been employed in a number of diagnostic assays. The MNPs are also capable of facilitating surface enhanced Raman scattering (SERS) enabling detection to become highly sensitive. Herein we demonstrate the expansion of the range of specific SERS-active oligonucleotide MNPs through the use of 12 new Raman-active monomethine and trimethine chalcogenopyrylium and benzochalcogenopyrylium derivatives. This has resulted in an increased ability to carry out multiplexed analysis beyond the current small pool of resonant and non-resonant Raman active molecules, that have been used with oligonucleotide functionalised nanoparticles. Each dye examined here contains a variation of sulphur and selenium atoms in the heterocyclic core, together with phenyl, 2-thienyl, or 2-selenophenyl substituents on the 2,2′,6, and 6′ positions of the chalcogenopyrylium dyes and 2 and 2′ positions of the benzochalcogenopyrylium dyes. The intensity of SERS obtained from each dye upon conjugate hybridisation with a complementary single stranded piece of DNA was explored. Differing concentrations of each dye (1000, 3000, 5000 and 7000 equivalents per NP-DNA conjugate) were used to understand the effects of Raman reporter coating on the overall Raman intensity. It was discovered that dye concentration did not affect the target/control ratio, which remained relatively constant throughout and that a lower concentration of Raman reporter was favourable in order to avoid NP instability. A relationship between the dye structure and SERS intensity was discovered, leaving scope for future development of specific dyes containing substituents favourable for discrimination in a multiplex by SERS. Methine dyes containing S and Se in the backbone and at least 2 phenyls as substituents give the highest SERS signal following DNA-induced aggregation. Principal component analysis (PCA) was performed on the data to show differentiation between the dye classes and highlight possible future multiplexing capabilities of the 12 investigated dyes.

## Abstract 2

12 new Raman active dyes are reported to increase the SERS intensity upon hybridisation of a targeted DNA to oligonucleotide-NP conjugates and can be potentially used together in a multiplex.

## Graphical abstract

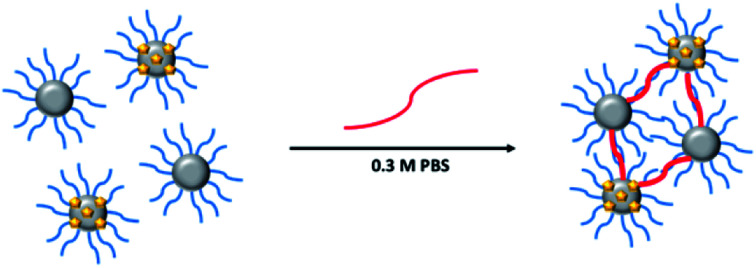

## Introduction

The detection and discrimination of DNA is of great importance as a diagnostic tool, especially in the identification of diseases caused by bacterial, fungal or viral infections. Currently, the most commonly used technique for the detection of DNA is quantitative polymerase chain reaction (qPCR) amplification coupled with fluorescence spectroscopy,^1,2^ however such methods can be time-consuming and often require expert handling due to the high risk of contamination. Alternatively, it is possible to design assays that use MNPs functionalised with oligonucleotides, which allow for sensitive detection using SERS when a single complementary strand of DNA is present (often termed the ‘target’).^3–10^ It has been demonstrated that SERS is a convenient technique because of its high sensitivity, non-destructive nature and its ability to simultaneously distinguish different targets in a multiplex.^11^ The great appeal of this technique can be attributed to the small linewidth of the molecularly specific vibrational Raman bands making the deconvolution of a multiplex spectrum easier.^8,12–19^

Mirkin *et al.*^20^ and Alivisatos *et al.*^21^ were the first to report the use of DNA for aggregating gold NPs in self-assembled structures. The aggregation between the MNPs occurred due to the Watson–Crick base-pairing between the oligonucleotide probes, attached to the MNPs surface through a thiol group, and a complementary target. Graham *et al.*^22^ and then Qian *et al.*^23^ used SERS to highlight the presence of a target DNA, taking advantage of the on/off SERS behaviour when coating the NPs surface with a Raman active dye. The presence of the dye at the surface of the NP is crucial as the DNA bases alone do not have significant Raman cross-sections. The base pairing and resultant NP aggregation is advantageous because it creates plasmonic hotspots. In these sites, the electromagnetic field is greatly enhanced due to the formation of clusters, which are responsible for the increase in Raman signal.^5,7,17,19,22–28^

Often the dyes associated with the NPs surface are both SERS active and fluorescent, these include dyes such as: 6-carboxyfluorescein (6-FAM),^10,26^ methylene blue,^26^ 5-carboxytetramethylrhodamine (TAMRA),^7^ TAMRA isothiocyanate (TRITC)^6^ and malachite green isothiocyanate (MGITC).^6,8^ In recently published research, a set of SERS active chalcogenopyrylium monomethine and trimethine dyes containing phenyl, 2-thienyl, and 2-selenophenyl substituents have been applied to hollow gold nanoshells^29,30^ and gold NPs.^31^ The binding to the gold surface most likely occurs through the chalcogen groups present, in this case selenium and sulphur, whilst the high levels of electron delocalisation in the molecules provide an intense SERS response.

These studies also highlighted that out of the two chalcogen atoms, selenium and sulphur, the former shows greater binding affinity to both gold and silver.^31–37^ It has been shown that the chalcogenopyrylium heteroatom rings are aromatic^37^ although the decrease in electronegativity^38^ and the increase in the atom and π orbitals size causes a less effective overlapping between the π orbitals of the heteroatom and the adjacent carbon atoms.^29,39,40^ The affinity of chalcogen atoms for silver, the aromatic properties and the high degree of conjugation of these chalcogenopyrylium dyes are interesting characteristics that make them good candidates as Raman reporters for use in DNA detection using SERS. However, to our knowledge no one has shown that they can be conjugated to the surface of NPs together with DNA in order to be used in SERS based assays. Establishing whether this is possible and if the hybridised system causes an increase in SERS signal is essential for the future design of DNA assays.

In this work oligonucleotide silver NPs conjugates have been coated with 12 new chalcogenopyrylium Raman-active dyes, in order to study DNA hybridisation to a target sequence with SERS and demonstrate the possible multiplexing capability of the 12 Raman reporters.

## Experimental

### Materials

Sodium hydroxide and sodium chloride were purchased from VWR (Leicestershire, UK). Hydroxylamine hydrochloride, silver nitrate, phosphate buffer powder, sodium citrate tribasic dihydrate and HPLC grade methanol were purchased from Sigma-Aldrich, (Dorset, UK). The water used throughout was doubly deionised.

**Fig. 1:**
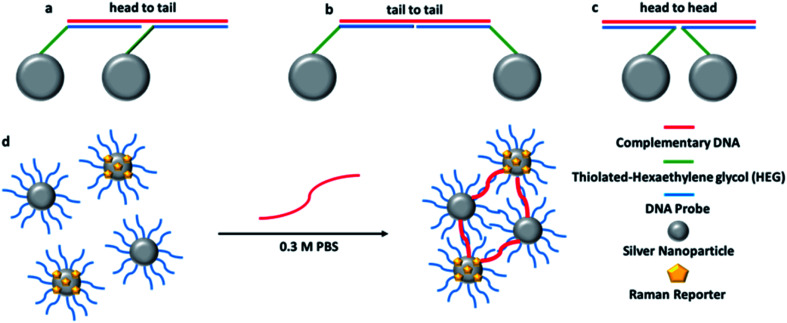
Schematic representation of each possible orientation of the oligonucleotide-NPs conjugates when hybridised to a complementary target sequence: (a) head to tail; (b) tail to tail; (c) head to head. A schematic representation of hybridisation of the conjugates in the presence of the target DNA is shown in (d).

The 12-mer DNA probes and the 24-mer target and control sequences were synthesised by ATDBio, (Southampton, UK). The probes were modified in the 5′ position with three thiol C6 chains followed by 3 hexaethylene glycol (HEG) chains. The probe sequences and complementary DNA were designed to hybridise in a head-to-tail conformation (shown in Fig. 1a).

**Fig. 2:**
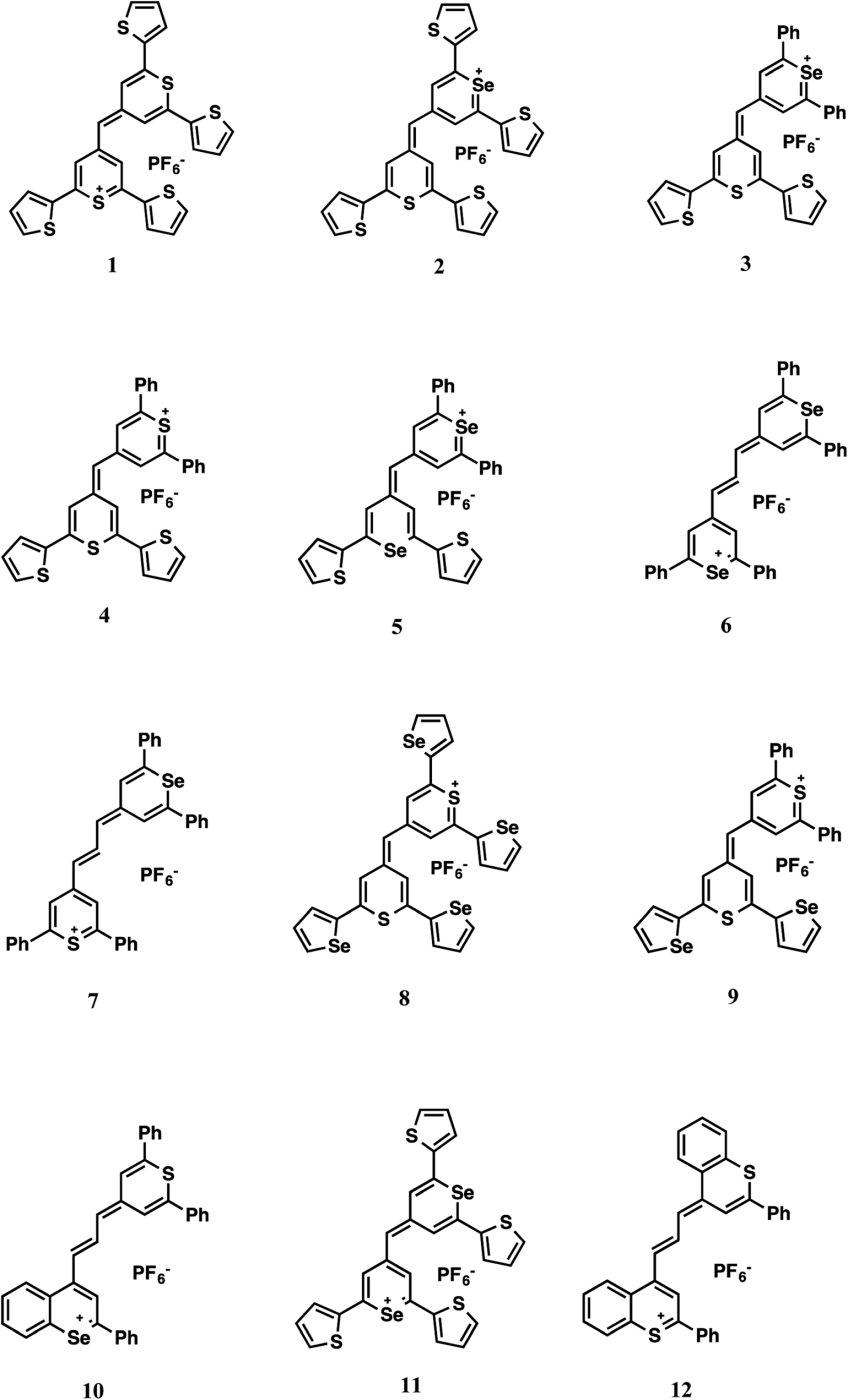
Dye structures.

**Scheme 1:**
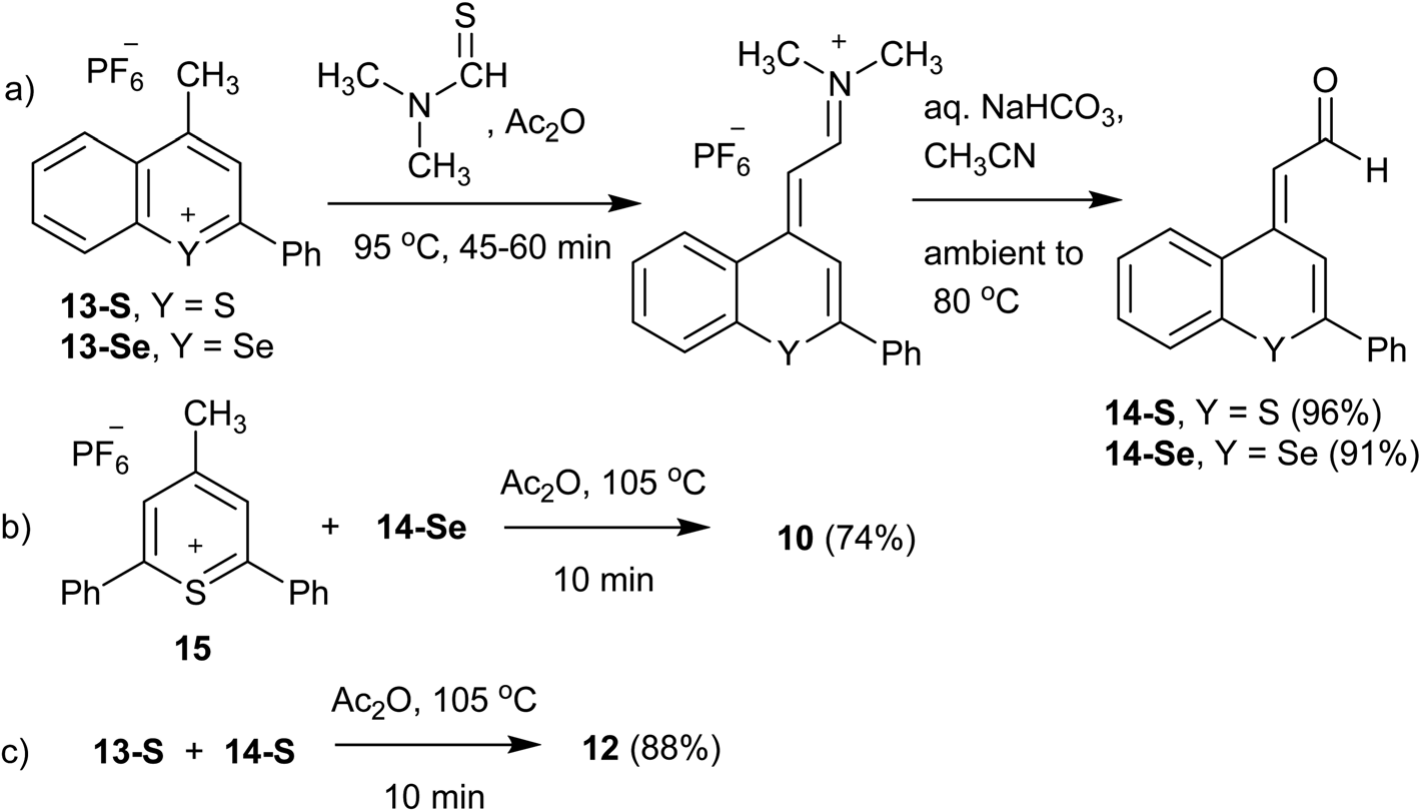
Synthesis of benzochalcogenopyrylium dyes 10 and 12: (a) synthesis of aldehydes 14-S and 14-Se; (b) synthesis of benzoselenopyrylium dye 10; (c) synthesis of benzothiopyrylium dye 12.

The Raman reporters 1–9 and 11 of Fig. 2 were synthesized according to a reported methodology.^29,31^ Trimethine benzochalcogenopyrylium dyes 10 and 12 were prepared by a related procedure as shown in Scheme 1. (Chalcogenobenzopyranylidene) acetaldehydes 14-S and 14-Se were prepared by treating a 4-methylbenzochalcogenopyrylium hexafluorophosphate salt 13-S^39^ and 13-Se^40^ respectively, with *N*,*N*-dimethylthioformamide in heated Ac_2_O to create the intermediate anilinium salts. Hydrolysis of the anilinium salts gave aldehydes 14-S and 14-Se with yields of 96% and 91%, respectively. Condensation of the aldehydes with either 4-methylthiopyrylium salt 15 or 13-S in Ac_2_O at 105 °C gave benzopyrylium dyes 10 (88%) and 12 (74%).

### Synthesis of 4-(3-(2,6-diphenyl-4*H*-thiopyran-4-ylidene)-prop-1-enyl)-2-phenylselenobenzopyrylium hexafluoro phosphate (10)

Thiopyrylium salt 15 (51.5 mg, 0.126 mmol), aldehyde 14-Se (43.2 mg, 0.139 mmol), and Ac_2_O (2.0 mL) were combined and heated at 105 °C for 10 min. The solution was cooled to ambient temperature, diluted with CH_3_CN, and the product precipitated with diethyl ether to yield hexafluoro phosphate 10 as a copper-bronze solid (65.3 mg, 74%). Mp > 260 °C, *λ*_max_ (CH_3_CN) = 748 nm (*ε* = 6.1 × 10^4^ M^−1^ cm^−1^). HRMS (ESI^+^) *m*/*z*: 557.0853 (calcd for C_35_H_25_S_80_Se^+^: 557.0837). Anal. calcd for C_35_H_25_SSe·PF_6_: C, 59.92; H, 3.59%. Found: C, 59.74; H, 3.48%.

### Synthesis of 2-phenyl-4-((*E*)-3-((*E*)-2-phenyl-4*H*-thiochrom-en-4-ylidene)prop-1-en-1-yl)-thiochromenylium hexafluorophosphate (12)

Benzopyrylium salt 13-S (65.1 mg, 0.170 mmol), aldehyde 14-S (54.0 mg, 0.204 mmol), and Ac_2_O (2.0 mL) were combined and heated at 105 °C for 10 min. The solution was cooled to ambient temperature, diluted with CH_3_CN, and the product precipitated with diethyl ether to yield hexafluoro phosphate 12 as a copper-bronze solid (94.4 mg, 88%). Mp 253–254 °C, *λ*_max_ (CH_3_CN) = 789 nm (*ε* = 1.5 × 10^5^ M^−1^ cm^−1^). HRMS (ESI^+^) *m*/*z*: 483.1240 (calcd for C_33_H_23_S_2_^+^: 483.1235). Anal. calcd for C_33_H_23_S_2_·PF_6_: C, 63.05; H, 3.69%. Found: C, 62.79; H, 3.88%.

### Synthesis of (*E*)-2-(2-phenyl-4*H*-thiochromen-4-ylidene)acetaldehyde (14-S)

4-Methyl-2-phenylthiochromenylium hexafluorophosphate 13-S (0.200 g, 0.524 mmol), *N*,*N*-dimethylthioformamide (0.134 mL, 1.57 mmol) and Ac_2_O (3.0 mL) were added to a round-bottom flask and heated at 95 °C for 45 min. After cooling to ambient temperature, the product was precipitated by addition of diethyl ether and cooling to −10 °C. The iminium salt was isolated by filtration, and hydrolysed by dissolving in CH_3_CN (3.0 mL), adding satd. aqueous NaHCO_3_ (3.0 mL) and heating the mixture to 80 °C over a 15 min period. The reaction mixture was maintained at this temperature for 30 min, after which it was diluted with H_2_O (50 mL) and the product extracted with CH_2_Cl_2_ (3 × 30 mL). The combined extracts were dried with MgSO_4_, and after concentration purified on SiO_2_ with first a CH_2_Cl_2_ eluent and then a 10% EtOAc/CH_2_Cl_2_ (*R*_f_ = 0.62) eluent to yield 14-S as a bright yellow crystalline solid (0.133 g, 96%). ^1^H NMR showed this to be a 95 : 5 mixture of the *E* : *Z* isomers, mp 91.0–92.5 °C. ^1^H NMR (*E* isomer) [500 MHz, CDCl_3_] *δ*: 10.27 (d, 1H, *J* = 6.5 Hz), 8.32 (s, 1H), 8.00 (d, 1H, *J* = 9.0 Hz), 7.70–7.68 (m, 2H), 7.51–7.42 (m, 6H), 6.49 (d, 1H, *J* = 6.5 Hz). ^13^C NMR [75.5 MHz, CDCl_3_] *δ*: 189.1, 146.2, 142.7, 137.2, 134.0, 130.1, 129.9, 129.1, 128.0, 127.2, 127.2, 126.7, 125.8, 117.1, 115.3. HRMS (ESI^+^) *m*/*z*: 264.0613 (calcd for C_17_H_12_OS: 264.0603).

### Synthesis of 2-(2-phenyl-4*H*-selenobenzopyran-4-ylidene)acetaldehyde (14-Se)

4-Methyl-2phenylselenobenzopyrylium hexafluorophosphate 13-Se (0.100 g, 0.233 mmol), *N*,*N*-dimethylthioformamide (59.4 μL, 0.698 mmol) and Ac_2_O (2.0 mL) were treated as described for the preparation of 14-S. This gave 14-Se as a yellow crystalline solid (66.0 mg, 91%). Mp 89–92 °C. ^1^H NMR [300 MHz, CDCl_3_] *δ*: 10.35 (d, 1H, *J* = 7.0 Hz), 8.34 (s, 1H), 8.00–7.97 (m, 1H), 7.65–7.57 (m, 3H), 7.49–7.40 (m, 5H) 6.53 (d, 1H, *J* = 6.5 Hz). ^13^C NMR [75.5 MHz, CDCl_3_] *δ*: 189.8, 148.4, 143.2, 138.9, 132.3, 129.9, 129.7, 129.12, 129.08, 128.1, 127.1, 126.9, 119.4, 119.1. HRMS (EI) *m*/*z*: 312.0037 (calcd for C_17_H_12_O_80_Se: 312.0048).

### Instrumentation

UV-Vis spectra were collected using a cell chamber Agilent Cary 300 Bio UV-visible spectrophotometer (Stockport, Cheshire, UK) with a spectral range from 200 to 800 nm and a resolution of 1 nm. Samples were transferred to quartz cuvettes with a 1 cm optical path for analysis.

Dynamic light scattering analyses were performed on a Malvern Zetasizer Nano ZS instrument (Worcestershire, UK). Measurements of hydrodynamic radius and zeta potential were performed to estimate the desired size, mono dispersion and charge of the synthesized colloid.

SERS analyses were performed on a RenDx SA-1000 (Renishaw Diagnostics Limited, Glasgow, UK) plate reader. The system equipped with a 532 nm laser was used to interrogate the samples using 2.5% laser power, 1 s exposure time for 1 accumulation. A spectral range from 0 to 4000 cm^−1^ and a resolution of 4 cm^−1^ was used. The *z*-axis distance between the plate and the beam source was optimised using EtOH as a standard.

### Colloid synthesis

Hydroxylamine silver colloid was synthesised by reduction of silver nitrate with hydroxylamine hydrochloride using a variant of the method first described by Leopold and Lendl.^41^

360 mL of a 0.235 M NaOH aqueous solution was prepared. 5 mL of aqueous 0.118 M hydroxylamine hydrochloride was added while stirring, followed by 40 mL of aqueous 9.86 mM AgNO_3_ after 2 min. The AgNO_3_ is added rapidly to minimise nucleation (a side effect that would result in a bigger distribution of the nanoparticles sizes).^41,42^ The mixture was then stirred at ambient temperature for 30 min. The synthesis was deemed successful when a yellow/green solution was formed.

### Conjugate synthesis

Typical synthesis of the conjugate involved the combination of 500 μL of as-synthesised nanoparticles with 5000 equivalents of an oligonucleotide probe. The sample was then allowed to shake at 200 rpm for 15 min before the addition of 0.250 M citrate buffer (40 μL, pH 2.9). After shaking for a further 15 min they were then centrifuged for 30 min at 8000 rpm. The supernatant was removed and the nanoparticle pellet was resuspended in a 0.1 M PBS buffer (500 μL, pH 7.4). NP concentration was determined using the *λ*_max_ and a reported extinction coefficient of 2.87 × 10^10^ mol^−1^ cm^−1^ given for 40 nm AgNPS.^43^

Dye solutions with a final concentration of 10^−5^ M in MeOH were prepared by dilution of DMF stock solutions and stored in the fridge. The dyes were added to 200 μL of the P2 conjugates in different amounts: 1000, 3000, 5000 and 7000 equivalents. After 45 min, the samples were centrifuged for 30 min at 8000 rpm, the supernatant was removed and 200 μL of 0.1 M PBS were added. It should be noted that the NP conjugates remain stable in this buffer for over a month. From this point on when the term P1 is used it refers to a silver nanoparticle with one of the two 12 base probes attached (complementary to one half of the target strand) and when the term P2 is used this refers to a nanoparticle with the second of the two probes attached plus one of the 12 dyes being studied.

### SERS samples

8 replicates for each sample, 4 samples using the target (complementary DNA), 4 samples using the control (non-complementary DNA), were prepared in PCR tubes up to a final volume of 150 μL. The sequences used are shown in Table 1.

**Table 1:** Oligonucleotide base sequence

Name	Sequence (5′-3′)
Model P1	TCTCAACTCGTA
Model P2	CGCATTCAGGAT
Target	TACGAGTTGAGAATCCTGAATGGC
Control	TCTCAACTCGTACGCATTCAGGAT

P1 and P2 conjugates have been added to reach a final concentration of 10 pM. 6 μL of 250 mM target and control aqueous solution were added, to reach a final concentration of 10 nM. Then the solutions were made up to a final volume of 60 μL with 0.3 M PBS (pH 7.4), following a methodology previously reported by Graham *et al.*^22^ After 20–30 min, the solutions were transferred to a 96-well plate, 90 μL of 0.3 M PBS were added and the SERS spectra were collected. In order to ensure the reproducibility of the study, samples were consistently analysed 30 min after the addition of target DNA (complementary and non-complementary).

## Results and discussion

Oligonucleotide conjugates NPs P1 and P2 have been designed to be each one half-complementary to a target DNA with a head to tail orientation, as shown in Fig. 1a. P2 conjugates have been coated with a Raman active dye, represented by yellow pentagons in Fig. 1d. The presence of the single stranded DNA target causes hybridisation to the NP conjugates. The base pairing between the sequences means that the NPs are transported into close proximity to each other creating hotspots and as a consequence the Raman intensity of the dye attached to P2 is increased. As the SERS spectrum is unique to the dye structure, it makes it possible to use different dyes attached to probe sets in order to simultaneously detect different targets within a multiplex.

For each dye, four replicate samples containing P1 and P2 conjugates, with target and then a control consisting of non-complementary DNA, were prepared. SERS spectra were collected, the intensities of the maximum peak of target and control were compared and the target/control ratios were calculated. The baseline corrected peak intensities used for the calculations were obtained manually by subtraction of underlying background signal from the total Raman peak. The same Raman shift (peak) was chosen for every spectrum when the same dye was used. The average values of SERS intensity for the four replicates are reported in Table 2 (dyes 1, 9 and 10) and Table 1s† (Dyes 2–8, 11 and 12).

**Table 2:** Samples data with dyes 10, 1 and 9. The Raman shift and Raman intensity maxima for the target and control spectra are reported, with the respective standard deviations calculated on the 4 replicates, at different concentrations of dye. In the last column are reported the on/off ratios

Dye no.	Dye equivalents	Peak position (cm^−1^)	Av comp SERS intensity (counts)	Av non-comp SERS intensity (counts)	Ratio (comp : non-comp)
10	1000	1607	91 ± 4	44 ± 6	2.06
	3000		139 ± 10	62 ± 7	2.25
	5000		179 ± 13	80 ± 15	2.24
	7000		245 ± 16	112 ± 13	2.18
1	1000	1316	199 ± 7	11 ± 12	1.79
	3000		415 ± 16	244 ± 13	1.70
	5000		538 ± 34	189 ± 31	1.86
	7000		630 ± 63	290 ± 20	2.17
9	1000	1607	917 ± 82	409 ± 57	2.24
	3000		1351 ± 101	660 ± 69	2.05
	5000		1513 ± 103	741 ± 56	2.04
	7000		1282 ± 134	596 ± 89	2.15

**Fig. 3:**
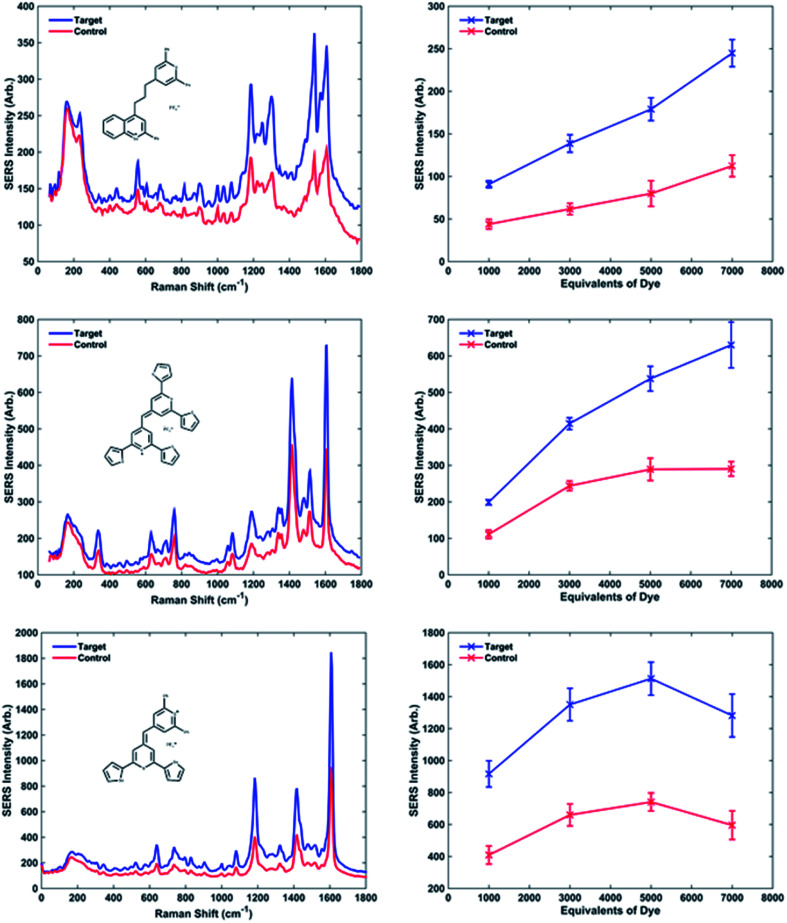
Examples of the three behaviours previously described. SERS spectra were recorded using an excitation wavelength of 532 nm, 1 1 s exposure time, 1 accumulation. The spectra obtained adding target and control to the NPs-DNA conjugates coated with 5000 equivalents of dye are compared using dye 10, 1 and 9 respectively. Raman intensity *vs.* concentration of dye are described showing a linear trend for dye 10, a plateau at high concentrations of dye meaning the saturation of the NPs for dye 1 and a maximum followed by a decrease in intensity for dye 9. The error bars reported are the standard deviations calculated for the 4 replicated of the analyses.

The intensities of the target and control peaks for each dye have been compared at each of the different dye concentrations (Fig. 3 and 1s†). This allowed the target/control ratios *vs.* concentration of dye to be studied. A range between 1000 and 7000 equivalents of dye per NP-DNA conjugate was chosen for investigation for every single dye. Throughout the experiments it was observed that some samples became unstable upon the addition of certain dyes (*e.g.* dye 2 after 3000 equivalents and dye 3 after 5000 equivalents displayed in Fig. 1s†); this highlighted the instability of the system as the concentration of the dye increases. Instability was demonstrated by a bathochromic shift and a decreased absorbance both of which were observed in the UV-Vis spectra (data not shown). The instability causes the nanoparticles to aggregate and is accompanied by a colour change from yellow to grey that is clearly observable by eye. It was noted during the collection of UV-Vis spectra that a small red shift (2–3 nm) was observed for every dye when the number of equivalents per nanoparticle was increased. The visible presence of large aggregates in the analyte solution became more evident when 7000 equivalents of dye were added. It was found, when comparing the methine with the trimethine dyes, that the SERS intensities of the maximum peak are generally higher for the methine dyes. The biggest difference between the structures is the planarity: the trimethine dyes should be co-planar, whilst the methine dyes should be twisted. This implies that a bigger electron cloud (up and down the plane of the entire molecule) is present in the trimethine dyes, and they would be theorised to have a bigger Raman intensity. Experimentally what we observed is exactly the opposite. A possible reason for that could be that if the dye aligns perpendicular to the NP surface, in the case of the trimethines any ring would be parallel to the metallic surface, whilst in the methine dyes the furthest ring of the backbone from the metal is possibly perpendicular to the NP, increasing the steric hindrance caused by the presence of the perpendicular DNA probes attached on the NP. A different orientation of the dye from the perpendicular would cause steric congestion. Trimethine dyes 7 and 10 were compared and showed very similar results. Both dyes contain a 2,6-diphenylthiopyrylium ring as a common structural feature and differ with a 2,6-diphenylselenopyrylium ring in 7 and a 2-phenylbenzoselenopyrylium ring in 10. The high similarity in the Raman intensity would suggest that the attachment to the surface occurs through the sulphur atom of the 2,6-diphenylthiopyrylium ring common to both dyes and that the Raman response is little affected by the differences between 2,6-diphenylselenopyrylium and 2-phenylbenzoselenopyrylium groups. This could be attributed to the longer distance from the surface attachment in the trimethine dyes. The backbones for methine and trimethine dyes have also been compared when common substituents were present (2 phenyl groups and 2 thienyl groups, then 4 thienyl groups with methine dyes; phenyl groups and condensed phenyl groups with trimethine dyes), showing in every case a higher SERS intensity when the backbone contains 1 S atom and 1 Se atom, followed by the dyes containing 2 S atoms and 2 Se atoms. Using different backbones in methine dyes, it has been observed in every case that the highest intensities are obtained when 2 phenyl substituents are present. This can be explained considering the higher stabilisation, and hence aromaticity of benzene, followed by thienyl and then selenophenyl groups.

The dyes which cause the highest SERS intensity in the spectrum are methine dyes containing S and Se in the backbone rings and at least 2 phenyl groups as substituents, as evidenced by the overall trends.

Observing graphs of SERS peak intensity *vs.* equivalents of dye, it is possible to distinguish different behaviours (Fig. 3 and 1s†). The curves relative to dyes 3, 5, 6 and 10 show a linear trend, between the increased dye concentration and resultant SERS intensity. The curves relative to dyes 1, 7 and 12 show a plateau at 3000 equivalents, meaning the saturation of the nanoparticle surface with dye occurs at high number of equivalents causing no more increase in the signal. Furthermore, dyes 4, 8, 9 and 11 have a maximum around 5000 equivalents followed by a decrease in intensity at 7000 equivalents. Although this phenomenon looks illogical, it can be explained by considering the non-specific aggregation caused by the high concentration of dye present, which causes the precipitation of larger particles, thus leaving much fewer NP conjugates suspended in solution and resulting in a less intense SERS plus the build-up of multi-layers of dye which reduce SERS signals.

The on/off ratios at different concentrations of dye can be considered almost constant; in fact their variation is minimal. Although in some cases the ratios are larger at a higher concentration of dye improving the overall sensitivity of the technique. The increase is not significant enough to risk non-specific aggregation therefore it would be always advisable to use fewer dye equivalents. From this work it has been concluded that 1000 equivalents are the best compromise between a consistent Raman signal and a non-specific aggregation.

### Multiplexing potential

**Fig. 4:**
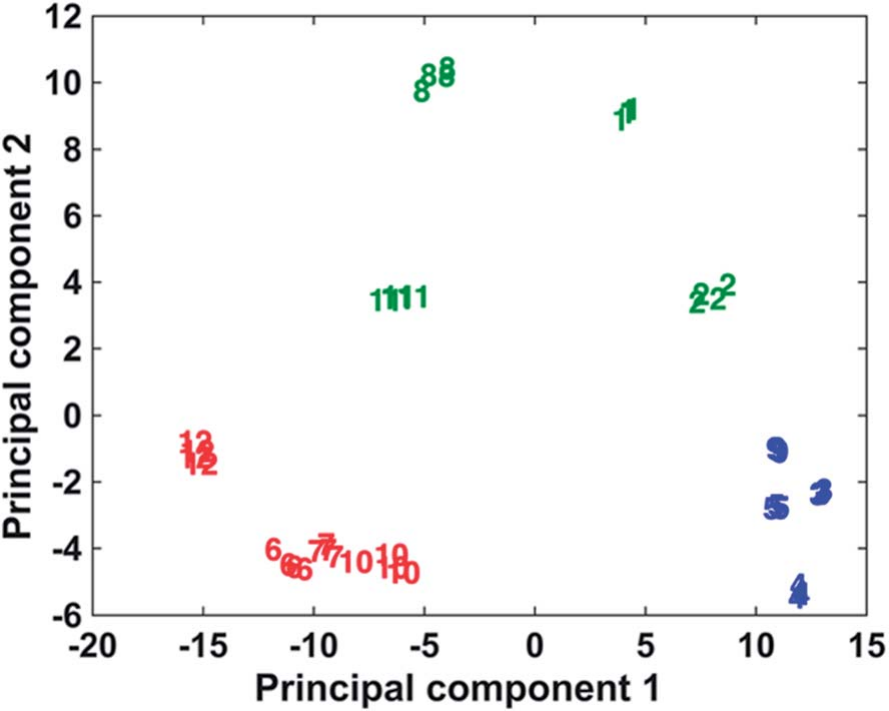
PCA plot demonstrating the discrimination of the 12 dyes into three classes. Monomethine dyes containing two phenyl groups as substituents (3–5 and 9) are shown in blue, methane methine dyes with four 2-thienyl or four 2-selenophenyl rings as substituents (1, 2, 8 and 11) are shown in green and trimethine dyes (6, 7, 10 and 12) are shown in red.

The ability to use the 12 dyes simultaneously to identify the presence of different DNA targets in a multiplex scenario was investigated using multivariate analysis in the form of PCA. PCA is a chemometric technique that reduces the dimensionality of data, thus making it easier to focus on the spectroscopic differences caused by the presence of different functional groups on the dyes structures.^44,45^ For the statistical analyses, the spectra obtained adding the complementary target to the conjugates coated with 3000 equivalents of dye have been used. Only the data relative to 1000 and 3000 equivalents of dye were present for every dye (some of the samples were unstable at higher concentrations of dye), and the use of the 3000 equivalents data seemed more opportune due to smaller signal/background ratio compared to the 1000 equivalents. The spectra were baseline corrected using a previously described method^46^ and autoscaled. The multivariate analysis was performed using Matlab R2014a, (MathWorks, Natick, MA, USA). As can be observed in Fig. 4, it is possible to differentiate the 12 dyes in 3 separate clusters. In the blue grouping are the monomethine dyes containing 2 phenyl groups as substituents: 3–5 and 9. These molecules showed the best behaviour as Raman reporters compared to the other dyes. The green group shows the methine dyes with four 2-thienyl or four 2-selenophenyl rings as substituents: 1, 2, 8 and 11. Finally, the red group includes all the trimethine dyes, 6, 7, 10 and 12, whose Raman response is the lowest observed compared to the methine dyes. All the dyes in the blue group produce very similar spectra and it is not possible to differentiate them unequivocally. A clear distinction can be made between all 4 dyes contained in the green group. Observing the red group, dye 12 is separated from dyes 6, 7 and 10, which are too similar to be uniquely distinguished. In conclusion, this shows the potential to identify up to the presence of 7 different targets using dyes 1, 2, 8, 12, a chosen dye between 6, 7 and 10 and a chosen dye between 3–5 and 9. The PCA results demonstrate the multiplexing capability of these dyes, whose similar structures have comparable SERS spectra. Table 2s† shows show the activity of the reporters correlates to the substituent groups.

## Conclusions

In conclusion, 12 chalcogenopyrylium and benzochalcogenopyrylium derivatives as monomethine and trimethine dyes containing sulphur and selenium and phenyl, 2-thienyl, and 2-selenophenyl substituents have been shown to be excellent SERS reporters when used in the creation of DNA-NP conjugates for the detection of target DNA. Different concentrations of dye have been used in order to understand how the amount of dye affected the Raman intensity and the on/off ratio. It has been demonstrated that 1000 dye molecules per NP is the optimal compromise between a high Raman intensity, a good on/off ratio, which is almost unvaried at different concentration of dye, and the non-specific aggregation. The highest intensities have been obtained with methine dyes containing 2 phenyl groups as substituents and a S and a Se atom in the backbone, whilst the lowest intensities were measured using the trimethine dyes. The multiplexing capability of the 12 dyes has been investigated performing PCA. Three distinct groups can be easily separated in the plot, corresponding to the trimethine dyes, the methine dyes containing 2 phenyl groups as substituents and the methine dyes substituted with 2-thienyl and 2-selenophenyl rings. 7 of 12 dyes produce Raman spectra sufficiently different to be distinguished in a PCA plot. This would suggest that they can be used to simultaneously detect 7 different targets. Future work will include the use of these dyes as Raman reporters coating bio-functionalized NPs for the detection of different biological targets as RNA, DNA, proteins, antibodies and enzymes.

## Conflicts of interest

There are no conflicts of interest to declare.

## Supplementary Material

RA-008-C8RA01998C-s001
