# Supplementary material for: Introducing 12 new dyes for use with oligonucleotide functionalised silver nanoparticles for DNA detection with SERS
Source: RSC Adv. 2018 May 15;8(32):17685–93. doi: 10.1039/c8ra01998c (PMC9080490; doi:10.1039/c8ra01998c)
Supplement: RA-008-C8RA01998C-s001 [file RA-008-C8RA01998C-s001.pdf]

Electronic Supplementary Material (ESI) for RSC Advances.
This journal is © The Royal Society of Chemistry 2018

Supplementary Information

Table 1s Samples data with dyes **2**, **3**, **4**, **5**, **6**, **7**, **8**, **9**, **11**, **12**. The Raman shifts and Raman intensity maxima for the target and control spectra are reported, with the respective standard deviations calculated on the 4 replicates, at different concentrations of dye. In the last column are reported the on/off ratios.

Dye N°	Dye Equivalents	Peak Position (cm^{-1})	Av Comp SERS Intensity (counts)	Av Non-Comp SERS Intensity (counts)	Ratio (Comp:Non-Comp)
**2**	1000	1603	651±57	267±16	2.44
	3000		1058±122	291±34	3.64
**3**	1000	1607	2010±211	675±84	2.98
	3000		3221±277	1024±115	3.15
	5000		4313±197	1588±91	2.72
**4**	1000	1611	1438±315	513±25	2.8
	3000		2009±156	636±38	3.16
	5000		3293±282	1670±142	1.97
	7000		2991±88	1650±63	1.81
**5**	1000	1607	1086±55	372 ± 41	2.92
	3000		2033±166	546 ± 58	3.73
	5000		2437±	598 ± 34	4.08
	7000		3691±248	1034 ± 86	3.57
**6**	1000	1603	66±6	31 ± 2	2.11
	3000		165±7	69 ± 4	2.41
	5000		222±9	93 ± 11	2.38
	7000		340±48	126 ± 4	2.7
**7**	1000	1607	132±3	58 ± 4	2.28
	3000		207±3	78 ± 11	2.64
	5000		324±23	130 ± 15	2.49
	7000		353±30	147 ± 15	2.4
**8**	1000	1425	104±8	82 ± 2	1.27
	3000		193±21	123 ± 9	1.56
	5000		208±20	127 ± 7	1.63
	7000		198±7	137 ± 16	1.45
**11**	1000	1603	79±6	23 ± 5	3.43
	3000		137±11	32 ± 6	4.35
	5000		347±36	85 ± 3	4.09
	7000		331±18	70 ± 12	4.77
**12**	1000	1316	20±4	12 ± 3	1.69
	3000		44±3	31 ± 1	1.44
	5000		117±5	77 ± 10	1.53
	7000		132±11	85 ± 8	1.55

Table 2s Here are reported the observed trends of Raman intensities varying the structure of the dye. The Raman intensities reported are those relative to 3000 equivalents of dye obtained adding the target, without being corrected.

Trend	Structure	Backbone	Substituents	Dye	SERS Intensities (counts)	Observations
1	Methane<br>trimethine	all<br>all	all<br>all	all<br>all	≥<br>≤	methine gave a higher intensity
2	trimethine	Se	2-phenyl selenochromene	**10**	288.07	Possible attachment through the S of the 2,2-phenyl thiopyryl ring
			2,2-phenil selenophenyl	**7**	285.45	
3	trimethine	S-S	2 condensed benzene, 2 phenyl	**12**	147.81	SERS Intensities<br>S-S > S-Se > Se-Se
			4 phenyl	**7**	285.45	
		S-Se	1 condensed benzene, 3 phenyl	**10**	288.07	
		Se-Se	4 phenyl	**6**	239.04	
4	methine	S-S	2 thienyl, 2 phenyl	**4**	2221.07	SERS intensity is bigger when there are 2 phenyls as substituents
			2 selenophenyl, 2 phenyl	**9**	1645.99	
			4 thienyl	**1**	576.50	
			4 selenophenyl	**8**	341.05	
		S-Se	2 thienyl, 2 phenyl	**3**	3550.32	
			4 thienyl	**2**	1222.73	
		Se-Se	2 thienyl, 2 phenyl	**5**	2255.49	
			4 thienyl	**11**	274.76	
5	methine	S-S	2 thienyl, 2 phenyl	**4**	2221.07	SERS Intensities<br>S-S > S-Se > Se-Se
		S-Se		**3**	3550.32	
		Se-Se		**5**	2255.49	
		S-S	4 thienyl	**1**	576.50	
		S-Se		**2**	1222.73	
		Se-Se		**11**	274.76	

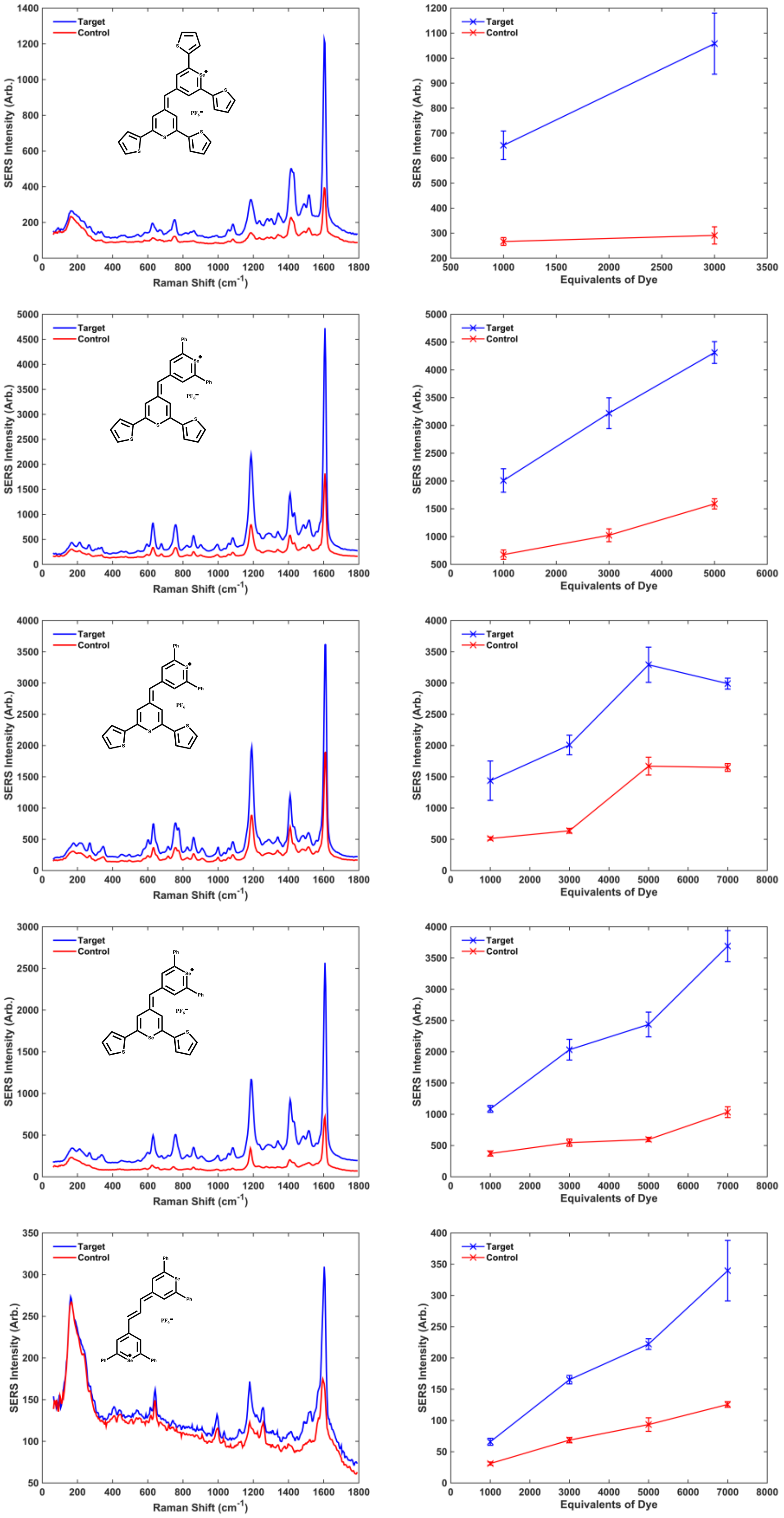

Target
Control
SERS Intensity (Arb.)
Raman Shift (cm^{-1})
Equivalents of Dye

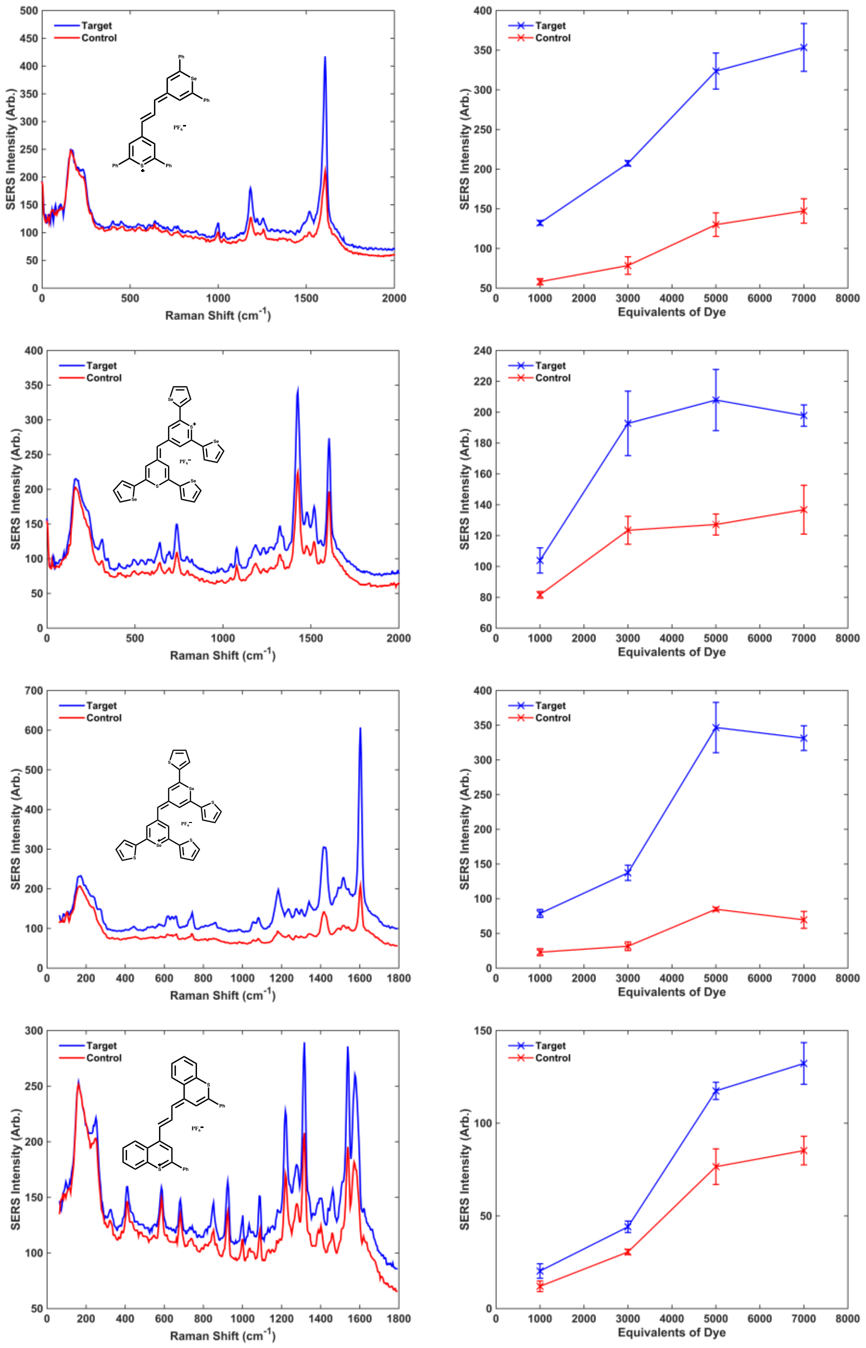

Figure 1s SERS spectra were recorded using an excitation wavelength of 532 nm, 1 s exposure time, 1 accumulation. SERS spectra adding target and control to the NPs-DNA conjugates coated with 5000 equivalents of dye are compared using dyes **3**, **4**, **5**, **6**, **7**, **8**, **11**, **12**, 3000 equivalents using dye **2**, on the left. Raman intensities *vs* concentrations of dyes are described on the right with dyes **2**, **3**, **4**, **5**, **6**, **7**, **8**, **11**, **12**. The error bars reported are the standard deviations calculated for the 4 replicated of the analyses.
